# Prevalence of *Mycoplasma genitalium* and *Mycoplasma hominis* in urogenital tract of Brazilian women

**DOI:** 10.1186/s12879-015-0792-4

**Published:** 2015-02-14

**Authors:** Guilherme Barreto Campos, Tássia Neves Lobão, Nathan Neves Selis, Aline Teixeira Amorim, Hellen Braga Martins, Maysa Santos Barbosa, Thiago Henrique Caldeira Oliveira, Djanilson Barbosa dos Santos, Tiana Baqueiro Figueiredo, Lucas Miranda Marques, Jorge Timenetsky

**Affiliations:** Instituto de Ciências Biomédicas, Departamento de Microbiologia, Universidade de São Paulo, Avenue Prof. Lineu Prestes nº1374 - Butantã, São Paulo, SP 05508-900 Brazil; Instituto Multidisciplinar em Saúde, Universidade Federal da Bahia, Rua Rio de Contas, 58, Quadra 17, Lote 58 Bairro Candeias Vitória da Conquista, Bahia, 45055-090 Brazil; Centro de Ciências da Saúde, Universidade Federal do Recôncavo Baiano, Av. Carlos Amaral, 1015 - Cajueiro, Santo Antônio de Jesus, BA CEP: 44.570-000 Brazil

**Keywords:** *Mycoplasma genitalium*, *Mycoplasma hominis*, urogenital infection

## Abstract

**Background:**

The role of *Mycoplasma hominis* and *M. genitalium* in urogenital tract infections remains unknown. Furthermore these mollicutes present a complex relationship with the host immune response. The role of inflammatory cytokines in infections also makes them good candidates to investigate bacterial vaginosis and mycoplasma genital infections. Therefore, the aim of this study was to detect the above-mentioned mollicutes by quantitative Polymerase Chain Reaction (qPCR) methodologies in vaginal swabs and dosage of cytokines.

**Methods:**

Vaginal swabs and peripheral blood were collected from 302 women, including healthy individuals. The molecular findings were correlated with some individual behavioral variables, clinical and demographic characteristics, presence of other important microorganisms in vaginal swabs, and levels of interleukin (IL)-1β and IL-6.

**Results:**

*M. hominis* and *M. genitalium* were detected in 31.8% and 28.1% of samples, respectively. The qPCR results were associated with clinical signs and symptoms of the infections studied. The frequency of *Trichomonas vaginalis*, *Gardnerella vaginalis*, *Neisseria gonorrhoeae* and *Chlamydia trachomatis* was 3.0%, 21.5%, 42.4%, and 1.7% respectively. Increased levels of IL-1β were associated with the presence of *M. hominis* and signs and/or symptoms of the genital infection of women studied.

**Conclusion:**

IL-1β production was associated with the detection of *M. hominis* by qPCR. The sexual behavior of women studied was associated with the detection of mycoplasma and other agents of genital infections.

**Electronic supplementary material:**

The online version of this article (doi:10.1186/s12879-015-0792-4) contains supplementary material, which is available to authorized users.

## Background

Sexually Transmitted Infections (STIs) occur worldwide and are an important public health problem. In developing countries STIs are among the five most frequent causes for seeking health services. The World Health Organization (WHO) estimates that about 340 million new cases of STIs occur each year. In Brazil, ten to twelve million new cases are reported each year [1,2].

Depending of the infectious agent, the STI is usually expressed in a skin lesion, secretion, vaginal discharge, wart, or blister. *Mollicutes* are included in STIs, but they are also found in healthy individuals. However five decades ago some mollicutes were considered infectious agents of the human urogenital tract [3]. This inconsistent history has made it challenging to clarify the role of these bacteria. The host-pathogen relationship is a complex and variable subject, but some interactions that have been studied have helped better understand this important relationship [4].

*M. hominis* is associated with non-gonococcal urethritis (NGU), bacterial vaginosis (BV) and post-birth fever [5], and rarely with bacteremia, arthritis [6], peritonitis [7] and meningitis [8]. In turn, *M. genitalium* was identified as a possible etiological agent of NGU and non-chlamydial urethritis [9]. This species has been detected in cervical samples from patients with salpingitis and acute endometritis [10]. Furthermore, *M. genitalium* has been strongly associated with cervicitis [11]. In women, an increase of vaginal discharge may be reported as well as dysuria, but the infection may be asymptomatic [12].

The mollicutes mentioned above also present a complex relationship in the host immune response [13]. It is well established that inflammatory cytokines play a critical role in regulating the response to infections. In fact these cytokine levels were associated with the presence of mycoplasmas. This renders them good candidates to be studied in the development of BV and mycoplasmal infections [14].

Although most genital mycoplasmas are cultured in a specific culture media, *M. genitalium* is quite fastidious. PCR assays for detecting this mollicute in clinical samples help better understand its incidence and distribution. In recent years, PCR became a readily-available and reliable method for detecting mollicutes in the human genital tract [15]. Therefore, the aim of this study was to detect *M. hominis* and *M. genitalium* and measure pro-inflammatory cytokines in a group of women with or without genital signs and/or symptoms of genital infection. The women studied live in Vitória da Conquista, a city located in the state of Bahia, Brazil.

## Results

*M. hominis* and *M. genitalium* were detected in 31.8% (96/302) and 28.1% (85/302), respectively in the women studied. The load of *M. hominis* and *M. genitalium* in the swab samples was measured by quantitative Polymerase Chain Reaction (qPCR). *M. genitalium* load ranged from 1 to ≥ 10^3^ copies/μL, while for *M. hominis,* it ranged from 1 to ≥ 10^4^ copies/μL. The mean amount of both species in swab samples was higher in women with signs of infection or symptoms than in the control group. However, no statistical difference was observed using the Mann–Whitney test.

The clinical samples were also tested by conventional PCR for other microorganisms. *T. vaginalis*, *N. gonorrhoeae*, *G. vaginalis* and *C. trachomatis* were found in 3.0%, 21.5%, 42.4%, and 1.7% of samples, respectively. Table 1 summarizes the co-infections detected with mycoplasmas and other species with gynecological importance.

**Table 1:** **Co-infection of genital microorganisms in vaginal samples of women from Vitória da Conquista, Brazil**

**Microorganisms**	**n (%)**
**MG + MH**	**15 (4.97)**
**MG + GV**	**12 (3.97)**
MG + CT	1 (0.33)
MG + NG	4 (1.32)
MG + MH + GV	6 (1.99)
MG + MH + NG	8 (2.65)
MG + MH + TV	1 (0.33)
MG + NG + GV	1 (0.33)
MG + MH + NG + GV	4 (1.32)
MG + MH + GV + TV	1 (0.33)
**MH + GV**	**21 (6.95)**
MH + NG	4 (1.32)
MH + TV	1 (0.33)
**MH + NG + GV**	**14 (4.63)**
MH + GV + CT	1 (0.33)
MH + TV + GV	1 (0.33)
MH + TV + NG + GV	1 (0.33)
GV + CT	2 (0.66)
**NG + GV**	**17 (5.62)**
**Total number of women with coinfection**	**115 (38.08)**

MG = Mycoplasma genitalium, MH = Mycoplasma hominis, GV = Gardnerella vaginalis, NG = Neisseria gonorrhoeae, CT = Chlamydia trachomatis, TV = Trichomonas vaginalis. N = 302 (absolute number of samples tested). The main co-infections found are marked in bold.

The *M. hominis* and *M genitalium* detected by qPCR using univariate analysis, showed that women under age 25 presented a higher risk for *M. hominis* infection (p = 0.034) [see Additional file 1], while those living in rural regions (p < 0.001), who sought consultation due to symptomatic reasons (p < 0.017), had one or more sexual partners in the last three months (p = 0.039), reported pruritus (p = 0.007) and presented vaginal discharge (p = 0. 006), presented a higher risk of infection to *M. genitalium* [see Additional file 1].

Using multivariate analysis, the risk for infection by *M. hominis* is associated with clinical visits due to symptoms, educational years (<12 years), age at first intercourse (≤15 years), sexually active, age (<25 years) and race/color (black/brown/indigenous) [see Additional file 1]. For *M. genitalium* infection, a highest risk was associated with region of residence, number of sexual partners in the past three months, having signs and/or symptoms suggestive of STI, and discharge and itchy genital site [see Additional file 1].

**Figure 1:**
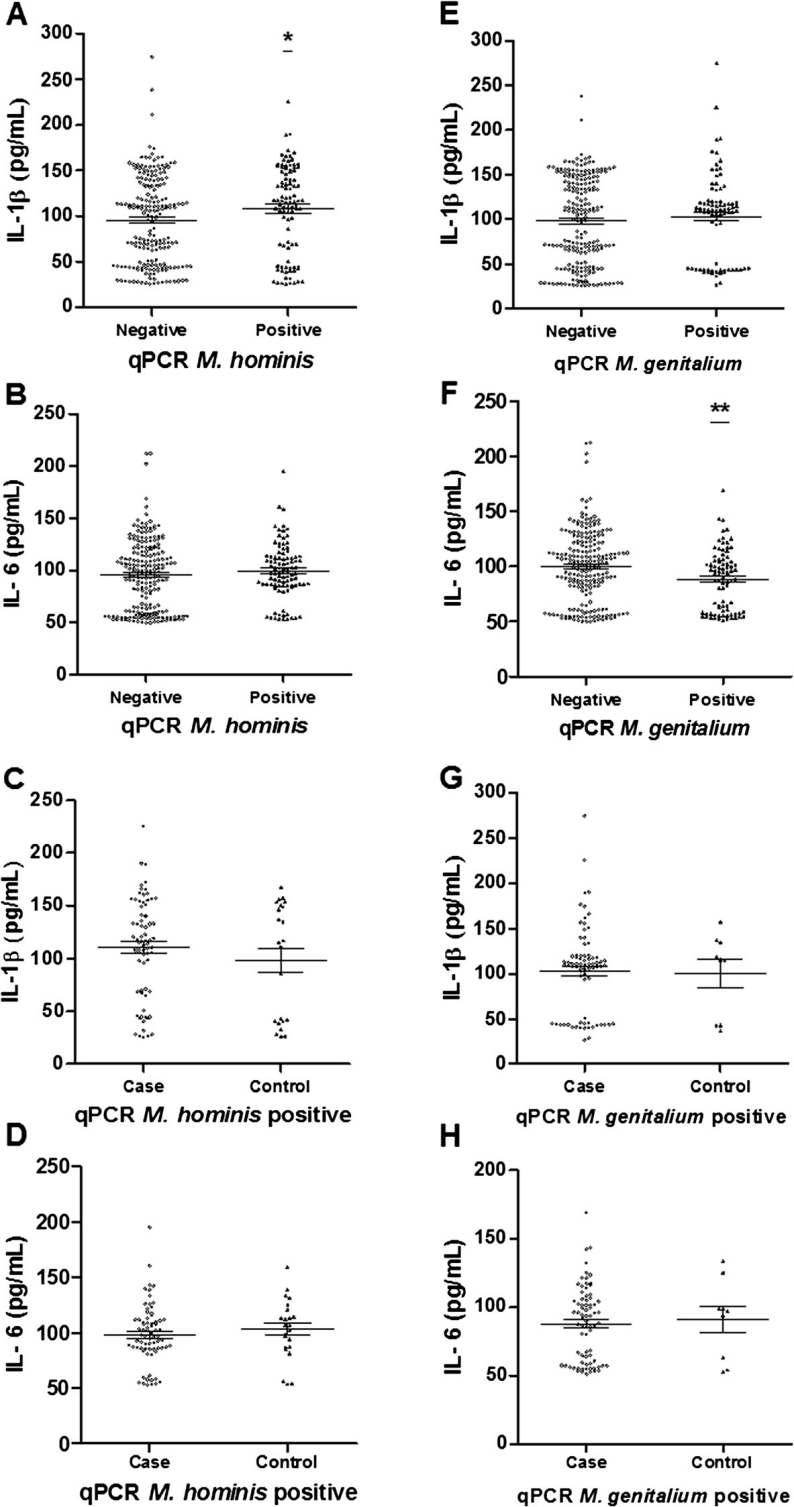
**Quantification of IL-1β and IL-6 (pg/mL) by ELISA in plasma samples. (A)** Concentration of IL-1β (pg/mL) in the plasma of groups of women qPCR positive and negative for *M. hominis*; **(B)** Concentration of IL-6 (pg/mL) in the plasma of groups of women qPCR positive and negative for *M. hominis* **(C)** Concentration of IL-1β (pg/mL) in plasma of women *M. hominis* qPCR-positive from case and control groups **(D)** Concentration of IL-6 (pg/mL) in plasma of women *M. hominis* qPCR-positive from case and control groups. **(E)** Concentration of IL-1β (pg/mL) in the plasma of groups of women qPCR positive and negative for *M. genitalium*; **(F)** Concentration of IL-6 (pg/mL) in the plasma of groups of women qPCR positive and negative for *M. genitalium* **(G)** Concentration of IL-1β (pg/mL) in plasma of women *M. genitalium* qPCR-positive from case and control groups **(H)** Concentration of IL-6 (pg/mL) in plasma of women *M. genitalium* qPCR-positive from case and control groups. Standard deviation and mean are indicated by solid lines in the graph. Statistical analysis by Mann Whitney. *p < 0.05; **p < 0.01.

Cytokine levels were compared between women qPCR-positive and negative for *M. hominis*. Women with *M. hominis* DNA presented a mean of interleukin (IL)-1β levels higher than those without *M. hominis* (p = 0.047) (Figure 1A). However, the IL-6 levels did not present a statistical difference between infected and non-infected women (p = 0.326) (Figure 1B). Furthermore, a statistical difference was not observed for IL-1β (p = 0.407) nor IL-6 levels (p = 0.332) (Figure 1C and 1D) when comparing case and control groups qPCR-positive for *M. hominis*.

Women with *M. genitalium,* otherwise presented IL-6 levels lower than the qPCR-negative group for this mollicute (p = 0.004) (Figure 1E). The IL-1β levels compared between the same two groups of women did not present a statistical difference (p = 0.833) (Figure 1F). Comparing cytokine levels between case and control groups qPCR-positive for *M. genitalium* also did not show a statistical difference in levels of IL-1β (p = 0.747) and IL-6 (p = 0.858) (Figure 1G and 1H).

## Discussion

In the present study, the frequency of *M. hominis* in the women studied was 31.8% using qPCR. There is still no consensus regarding the role of detected *M. hominis*, although *M. hominis* has been isolated from urogenital infections. However, the presence of *M. hominis* (MH) has been correlated with the development of pelvic inflammation, spontaneous abortions and infertility [16]. Despite the lack of statistical significance, the average MH load was higher in women with signs and/or symptoms than without this condition. Probably the bacterial load is closely related to the development of the disease. Few studies have reported the detection of MH by qPCR. Baczynska et al. [17] observed the presence of MH using qPCR in only 2.4% of cervical samples of women attending fertility clinics in Denmark. According to the authors, the results were similar when compared with culture results. Likewise, Cunningham et al. [18] obtained positive findings for MH using qPCR in 14% of genitourinary samples (vaginal swabs and urine) in the United States. Most studies focus on conventional PCR analysis [19]. In southeastern Brazil, Rodrigues et al. [20], in a study of cervical swabs of 224 women who attended three specific women’s healthcare clinics, reported MH in 21.9% of samples.

In the present study, we observed that first intercourse before age 15, and age less than 25 were associated with the risk of infection by MH. Similar results were reported by Kataoka et al. [21] who analyzed 877 samples of vaginal swabs in Japan and Verteramo et al. [22] who analyzed the risk behavior of women infected with MH. No association was observed for other STI markers. However, a study performed with 153 Greek women, reported a strong association of MH with BV, urinary symptoms (e.g. unexplained chronic voiding, dysuria and urethritis) and active sexual activity [23]. Furthermore, other positive associations were also reported in the literature such as *C. trachomatis* co-infection [21], *G. vaginalis*, *T. vaginalis* and *U. urealyticum* co-infections [24], abortion [22], use of condoms, race or multiple sexual partners [24].

*M. genitalium* (MG) is recognized as an etiological agent of NGU in men, while in women it is recognized as an etiological agent of cervical inflammation, infertility and pelvic inflammatory disease. Prevalence of 28.1% (85/302) was found in this study using qPCR. Normally the prevalence in the population of MG is considered low. Falk et al. [25], using urine and cervical samples of women attending an STD clinic for women in Orebro, Sweden, reported frequency of 6%. Walker et al. [26], in a cohort study of 16 to 25 year old Australian women recruited from primary healthcare clinics, also observed a low frequency of MG (2.4%). In Brazil, Rodrigues et al. [20] reported a frequency of only 0.9%. However, Casin et al. [27] observed a high prevalence of MG in women attending a sexually-transmitted-disease clinic in Paris. In addition, MG was recovered more often from the vaginal epithelial cells (39%) than from the cervix (21%). Similar results in women attending sexually-transmitted-disease clinics were also observed by Gaydos et al. [28] and Mobley et al. [29] who reported a prevalence of 19.2%. Furthermore, Mobley et al. [29] also detected a higher prevalence of MG in vaginal samples. According to the authors, this may reflect a minimal host immune response to MG infection in the vaginal epithelium, and thus greater bacterial survival in the vagina compared to the endocervix, where the inflammatory response to MG by endocervical epithelial cells is more robust. Herein the load of MG in the studied samples was also higher in symptomatic than in asymptomatic women. However, there was no significant statistical difference in both groups. Among the women studied with signs and symptoms of STI, 32.2% were qPCR positive for MG; the relation between these two events was statistically significant (p = 0.004) (data not shown).

In the present study, several risk factors for mycoplasmal infection were analyzed in studies using logistic regression. The highest risk was associated with the Brazilian region of residence, number of sexual partners in the past three months, having signs and/or symptoms suggestive of STI, including runny discharge and itchy genital site. The risk of infection for MG associated with the age of women is variable in the literature. Consistent with the present study, Huppert et al. [30] and Kataoka et al. [21] also observed no relation between the ages of women. No mention of positive association was observed between signs and symptoms and MG infection in other studies. On the contrary, a negative association with the symptom dysuria has been reported [29]. In addition, the following risk factors have been reported in the literature for MG urogenital infection in women, including: a recent sexual contact [30], premature birth [31], number of sexual partners in the last month, smoking, intimate hygiene with douche and history of spontaneous abortion [32]. Coinfection with *C. trachomatis* and *T. vaginalis* [31] were also reported as risk factors for MG infection.

The detected frequencies for *T. vaginalis*, *N. gonorrhoeae*, *G. vaginalis* and *C. trachomatis* in women studied were 3.0%, 21.5%, 42.4%, and 1.7% respectively. The difference in prevalence between the mycoplasmas and the searched microorganisms and risk factors compared with the literature could be related to the population studied. Some authors confirm that the prevalence of genital microorganisms is related to regional differences [1,27,28,33-35]. However, explanations for these differences are not clear, are variable and require more study. The women studied live in the Northeast region of Brazil, which has a semi-arid climate, with high rates of poverty. This population depends strictly on basic governmental health services. At the time of collection of clinical material, the healthcare service for women was not in service for several months. We believe this factor associated with lack of knowledge regarding prevention and treatment of STIs may have contributed to this prevalence obtained.

In addition, approximately thirty-eight percent of women showed colonization with more than one microorganism. The most common coinfections were between *M. hominis* and *G. vaginalis* (6.95%). These two microorganisms are considered commensal of the genital tract [36]. Leli et al. [37] observed that patients positive for *M. hominis* were more frequently colonized by *G. vaginalis*. The coinfection of *G. vaginalis* and *N. gonorrheae* (5.62%), *M. genitalium* and *M. hominis* (4.97%), *M. hominis*, *N. gonorrheae* and *G. vaginalis* (4.63%), and *M. genitalium* and *G. vaginalis* (3.97%) also were observed. The coinfection among genital microorganisms has been frequently reported [20,26,28,29,38,39]. According Gaydos et al. [28], these findings have important implications in the diagnosis and treatment of genital infections. There are no treatment guidelines for genital mycoplasmas in Brazil. The antibiotics used are not standardized and may not be effective in all cases of *M. genitalium* or *M. hominis* infection.

The opportunistic role of mollicutes includes some features of the innate immune response. In the present study, a higher level of IL- 1β was observed in infected women than in non-infected with MH, but no difference was observed in IL-6 levels*.* In the literature, the presence of MH was associated with higher levels of vaginal IL-1β, but not with vaginal interleukin-1 receptor antagonist (IL-1rα) levels nor intra-amniotic fluid levels of IL-1β, IL-1rα and IL-6 [40]. According to Wasiela et al. [41], vaginal levels of IL- 8 but not IL-1α, IL-1β or IL-6 are higher in women with mycoplasma infection. Ryckman et al. [14] observed that vaginal IL-1β, IL-6 and IL-8 levels in pregnant were significantly higher in women with any mycoplasma compared to those without. This may be related to bacterial pathogen-associated molecular pattern (PAMPS). *Mollicutes* possess a large number of lipoproteins, termed lipid-associated membrane proteins (LAMPs). Recognition of LAMPs by the innate immune system can trigger the production of various proinflammatory cytokines from manifold cells, a surface component that causes inflammation [42-44]. Therefore it is possible to conclude that the immune response to mollicutes is variable due the host and the antigenic mosaic and variation of mollicutes. This may explain in part the potential role of *Mollicutes* in mammals to be excellent opportunistic microorganisms that modulate the immune response differently.

Women infected with MG showed mean levels of IL-6 lower than the qPCR-negative group. These findings may be explained by the low *M. genitalium* load, or persistent infection. In fact, IL-6 is produced mainly in the acute phase of inflammation in the vagina [45]. McGowin et al. [46] found that MG induce IL-6 in human A2EN and ShEN101 cells during acute secretion (48 h). However, in a persistent infection lasting 36 days, a noted, but insignificant level of IL-6 was detected. Furthermore, the interleukin concentrations could be lower in plasma, despite the presence of this microorganism.

## Conclusions

This study also contributed to the epidemiological data of STI in the chosen geographic region for local public health, especially because most STIs in Brazil are not notifiable diseases. Due to the complexity of vaginal microbiota and limited understanding of its role in urogenital diseases, further studies of mollicutes are needed to better understand the role of these bacteria in the urogenital tract of women.

## Methods

### Women studied

This analytical cross-sectional study analyzed clinical samples from Vitória da Conquista city, the third largest city of Bahia State, in the northeast of Brazil. The women included in the study attended a Brazilian public health clinic, known as the ‘Unified Health System’. The study population consisted of 302 women, ages ranging from 14 to 78 years, and averaging 37 years. Clinical samples were obtained (vaginal swab and peripheral blood). Each woman was asked a series of questions and their answers were recorded. The exclusion criteria were pregnancy, HIV positive and with antimicrobial therapy in the last 3 months. Clinical signs and symptoms, such as discharge, itch, fetid odor and dysuria were considered. Furthermore, information about sexual experience and behavior were gathered in interviews with trained staff to minimize information bias.

### Ethics statement

The study protocol was approved by the Research Ethics Committee of the Biomedical Science Institute of São Paulo University (998/2012). Study subjects provided written informed-consent before participating in the study

### Clinical samples

The samples were collected from May to July of 2011 and January of 2012. The swabs were rubbed on the internal surface of vagina. The samples were stored at 4°C in 5 mL of transport media. Blood samples were collected in two 5 mL tubes with and without Ethylenediamine tetracetic acid (EDTA) and the serum was stored at −20°C for Enzyme Linked Immuno Sorbent Assay (ELISA) assay. The vaginal samples were homogenized and stored at −20°C for DNA extraction.

### Quantitative PCR (qPCR)

Genomic DNA samples of vaginal swabs were obtained according to the recommendations of Purelink™ Genomic DNA Mini Kit (Invitrogen, São Paulo, SP, Brazil). Real-time PCR was performed in duplicate for each sample. A protocol for DNA amplification was performed with a TaqMan Probe for *M. hominis* [47] and *M. genitalium* [48]. For constructing DNA standards for absolute quantitation, the mycoplasmas were first cultured in 2 ml at 37°C and expanded to a 50 ml of SP4 broth. In a logarithmic growth phase (based in colorimetric changes), the culture was centrifuged at 20,600 g for 30 minutes at 25°C. The DNA was extracted using a PureLink™ Genomic DNA Mini Kit. The genomic DNA copy number was then calculated by spectrophotometry (NanoDrop ND-1000, Witec Ag, Littau, Switzerland). Ten-Fold serial dilutions (10^7^–10 copies/μl) of the mycoplasma DNA standard were prepared and analyzed.

Differential conventional PCR was also performed to detect *G. vaginalis* [49], *T. vaginalis* [50], *N. gonorrhoeae* and *C. trachomatis* [51].

### Cytokine quantification

An ELISA kit (Bioscience, San Diego, C. A., United States) and high-absorption 96-well microplates were used to quantify cytokines. After standardization of a curve and volume sample, 100 μL of each serum was used to quantify cytokines IL-1β and IL-6. The immunoenzymatic reactions were measured by ELISA with a wavelength of 450 nm.

### Statistical analysis

Clinical and epidemiological data obtained were analyzed using the SPSS 16.0 software (SPSS Inc., Chicago, USA). The association between variables was compared applying the chi-square Pearson test or Fisher’s exact test. Statistical significance was considered p < 0.05 with a 95% CI. Risk factors associated with infection were evaluated by the Odds Ratio (OR) and univariate analysis. All variables with p < 0.10 were included in multivariate analysis using logistic regression. Multivariate logistic regression was used to assess the independent contribution of the variables associated with the detection of microorganisms. For cytokine quantification data analysis, the GraphPad Prism program was used. The nonparametric Mann–Whitney test was used when evaluating two groups.

## Additional file

Additional file 1:
**Univariate and multivariate analysis of possible risk factors for** ***Mycoplasma hominis*** **and** ***M. genitalium*** **infections in women from Vitória da Conquista, Brazil, 2011.**
