# Supplementary material for: Prevalence of Mycoplasma genitalium and Mycoplasma hominis in urogenital tract of Brazilian women
Source: BMC Infect Dis. 2015 Feb 14;15:60. doi: 10.1186/s12879-015-0792-4 (PMC4336719; doi:10.1186/s12879-015-0792-4)
Supplement: Additional file 1: — Univariate and multivariate analysis of possible risk factors for Mycoplasma hominis and M. genitalium infections in women from Vitória da Conquista, Brazil, 2011. [file 12879_2015_792_MOESM1_ESM.doc]

**Additional file 1.** Univariate and multivariate analysis of possible risk factors for *Mycoplasma hominis* and *M. genitalium* infections in women from Vitória da Conquista, Brazil, 2011.

| **Variables** | ***M. hominis***  **Positive** | | ***M. hominis***  **Negative** | **Odds Ratio**  **IC 95%** | **Adjusted**  **Odds Ratio** | ***M. genitalium***  **Positive** | ***M. genitalium***  **Negative** | **Odds Ratio**  **IC 95%** | **Adjusted Odds Ratio** |
| --- | --- | --- | --- | --- | --- | --- | --- | --- | --- |
|  | **N = 96** | **N = 206** | |  |  | **N = 85** | **N = 217** |  |  |
|  | **n (%)*** | **n (%)†** | |  |  | **n (%)*** | **n (%)†** |  |  |
| **Demographic data** |  |  | |  |  |  |  |  |  |
| **Region**  Rural  Urban | 21 (21.9)  75 (78.1) | 51 (24.8)  155 (75.2) | | 0.85 [0.47-1.51] | –‡ | **38 (44.7)**  **47 (55.3)** | **34 (15.7)**  **183 (84.3)** | **4.35 [2.47-7.64]^a^** | **3.3 [1.8-6.1]** |
| **Reason for visit^5^**  Symptomatic  Routine | 30 (31.9)  64 (68.1) | 56 (27.5)  148 (72.5) | | 1.23 [0.72-2.10] | –‡ | **32 (39.0)**  **50 (61.0)** | **54 (25.0)**  **162 (75.0)** | **1.29 [1.11-3.29]^b^** | **1.3 [0.7-2.4]** |
| **Age**  < 25 years  ≥ 25 years | **23 (24.0)**  **73 (76.0)** | **29 (14.1)**  **177 (85.9)** | | **1.92 [1.04-3.54]^b^** | **1.7 [0.9-3.2]** | 15 (17.6)  70 (82.4) | 37 (17.1)  180 (82.9) | 1.04 [0.53-2.01] | –‡ |
| **Race**  Black /Indigenous  Caucasian/Asian | 78 (81.2)  18 (18.8) | 148 (71.8)  58 (28.2) | | 1.69 [0.93-3.08] | 1.6 [0.9-3.0] | 66 (77.6)  19 (22.4) | 160 (73.7)  57 (26.3) | 1.23 [0.68-2.23] | –‡ |
| **Education, years**  < 12  ≥ 12 | 73 (76.8)  22 (23.2) | 132 (68.8)  60 (31.2) | | 1.50 [0.85-2.65] | –‡ | 61 (72.6)  23 (27.4) | 144 (70.9)  59 (29.1) | 1.08 [0.61-1.91] | –‡ |
| **Stable Relationship**  No  Yes | 24 (25.0)  72 (75.0) | 39 (18.9)  167 (81.1) | | 1.47 [0.80-2.54] | –‡ | 17 (20.0)  68 (80.0) | 46 (21.2)  171 (78.8) | 0.92 [0.49-1.73] | –‡ |
| **Sexual Health** |  |  | |  |  |  |  |  |  |
| **Menarche**  < 15 years  ≥ 15 years | 71 (74.0)  25 (26.0) | 167 (81.1)  39 (18.9) | | 0.66 [0.37-1.17] | –‡ | 68 (80.0)  17 (20.0) | 170 (78.3)  47 (21.7) | 1.10 [0.59-2.06] | –‡ |
| **First Sexual Intercourse**  ≤ 15 years  > 15 years | 28 (29.2)  68 (70.8) | 44 (21.4)  162 (78.6) | | 1.51 [0.87-2.63] | –‡ | 21 (24.7)  64 (75.3) | 51 (23.5)  166 (76.5) | 1.06 [0.59-1.91] | –‡ |
| **Sexual Activity^1^**  Active  Inactive | 92 (95.8)  4 (4.2) | 183 (89.3)  22 (10.7) | | 2.76 [0.92-8.26] | 2.5 [0.8-7.7] | 81 (95.3)  4 (4.7) | 194 (89.8)  22 (10.2) | 2.29 [0.76-6.87] | –‡ |
| **Libido**  Decreased  Maintained | 43 (45.3)  52 (54.7) | 91 (44.6)  113 (55.4) | | 1.02 [0.63-1.67] | –‡ | 36 (42.9)  48 (57.1) | 98 (45.6)  117 (54.4) | 0.89 [0.53-1.48] | –‡ |
| **Sexual partners in life**  ≥ 5  < 5 | 9 (9.4)  87 (90.6) | 18 (8.8)  187 (91.2) | | 1.07 [0.46-2.48] | –‡ | 10 (11.8)  75 (88.2) | 17 (7.9)  199 (92.1) | 1.56 [0.68-3.56] | –‡ |
| **Sex partners last 3 months**^§^  ≥ 1  None | 87 (91.6)  8 (8.4) | 175 (85.8)  29 (14.2) | | 1.80 [0.79-4.10] | –‡ | **78 (94.0)**  **5 (6.0)** | **184 (85.2)**  **32 (14.8)** | **2.71 [1.01-7.22]^b^** | **1.7 [0.6-4.9]** |
| **Condom use**  Rare/Occasional  Always | 80 (86.0)  13 (14.0) | 171 (85.5)  29 (14.5) | | 1.04 [0.51-2.11] | –‡ | 70 (85.4)  12 (14.6) | 181 (85.8)  30 (14.2) | 0.96 [0.46-1.99] | –‡ |
| **Hormonal contraception**  Yes  No | 32 (34.0)  62 (66.0) | 78 (38.4)  125 (61.6) | | 0.82 [0.49-1.38] | –‡ | 34 (40.5)  50 (59.5) | 76 (35.7)  137 (64.3) | 1.22 [0.73-2.05] | –‡ |
| **Dyspareunia**^2^  Yes  No | 28 (30.1)  65 (69.9) | 64 (32.2)  135 (67.8) | | 0.90 [0.53-1.54] | –‡ | 24 (28.9)  59 (71.1) | 68 (32.5)  141 (67.5) | 0.84 [0.48-1.47] | –‡ |
| **Post coital Bleeding^3^**  Yes  No | 9 (9.5)  86 (90.5) | 17 (8.6)  181 (91.4) | | 1.11 [0.47-2.60] | –‡ | 8 (9.6)  75 (90.4) | 18 (8.6)  192 (91.4) | 1.13 [0.47-2.72] | –‡ |
| **Dysuria**  Yes  No | 17 (17.7)  79 (82.3) | 47 (22.8)  159 (77.2) | | 0.72 [0.39-1.34] | –‡ | 20 (23.5)  65 (76.5) | 44 (20.3)  173 (79.7) | 1.21 [0.66-2.20] | –‡ |
| **Pelvic pain**  Yes  No | 43 (44.8)  53 (55.2) | 92 (44.7)  114 (55.3) | | 1.01 [0.61-1.63] | –‡ | 43 (50.6)  42 (49.4) | 92 (42.4)  125 (57.6) | 1.39 [0.84-2.30] | –‡ |
| **Itch**  Yes  No | 27 (28.1)  69 (71.9) | 63 (30.6)  143 (69.4) | | 0.88 [0.52-1.51] | –‡ | **35 (41.2)**  **50 (58.8)** | **55 (25.3)**  **162 (74.7)** | **2.06 [1.21-3.50]^b^** | **1.6 [0.9-3.0]** |
| **Discharge^4^**  Yes  No | 43 (45.3)  52 (54.7) | 91 (44.2)  115 (55.8) | | 1.04 [0.64-1.70] | –‡ | **48 (57.1)**  **36 (42.9)** | **86 (39.6)**  **131 (60.4)** | **2.03 [1.21-3.38]^b^** | **1.5 [0.8-2.8]** |

Missing observations: ^1^sexual life: loss of 1 (n=301); ^2^dyspareunia: 10 dropouts and excluded women in inactive sexual life (n=292); ^3^postcoital bleeding: 9 dropouts and excluded women with inactive sexual life (n=293); ^4^discharge: loss of 1 (n=301); ^5^reason of visit: loss of 4 (n=298).

*****Percentage of total study population with characteristic testing positive for *M. hominis* and *M. genitalium* by qPCR.

†Percentage of total study population with characteristic testing negative for *M. hominis* and *M. genitalium* by qPCR.

§Sexual partners in the last 3 months.

‡Variables with a p-value greater than 0.10 were not included in the multivariate analysis.

CI, confidence interval

^a^ *P* < .001.

^b^ *P* < .05
